# Teaching the Holocaust in Nursing Schools: The Perspective of the Victims and Survivors

**DOI:** 10.3390/ijerph18178969

**Published:** 2021-08-26

**Authors:** Zvika Orr, Anat Romem

**Affiliations:** Department of Nursing, Jerusalem College of Technology, Jerusalem 9548311, Israel; romem@g.jct.ac.il

**Keywords:** ethics, Holocaust studies, Holocaust survivors, leadership, nurses, nursing education, post-trauma, reflection, resilience, total institutions

## Abstract

In recent years, there has been increased recognition of the significance and relevance of Holocaust studies to nurses. However, these studies are rarely integrated in the nursing curriculum, and even when they are, the focus is usually on healthcare personnel who collaborated with the Nazi regime. This article aims to bridge this gap by analyzing a comprehensive requisite curriculum on the Holocaust for graduate nursing students. We emphasize the work of Jewish healthcare professionals during the Holocaust and the dilemmas they faced, as well as the trauma and resilience of Holocaust survivors, their treatment today, and implications for treating other patients. This article examines how studying these issues affected the graduate students. It analyzes the reflective accounts written by the students, using qualitative content analysis and Grounded Theory. The findings suggest that students received tools to act professionally and empathetically while demonstrating greater sensitivity to the patients’ identity, past experiences, trauma, and how the hospital as a “total institution” affects them. Many of the students developed conscious leadership. The program used a personalized pedagogical approach that contributed to experiential learning but was also emotionally challenging for the participants. We recommend including Holocaust studies as a requisite component in nursing programs worldwide.

## 1. Introduction

The Holocaust provides many lessons in medical ethics. Lessons can be learned from the horrific violation of human rights by Nazi doctors and nurses, which included medical experiments on prisoners, forced sterilization, and “euthanasia” [1,2]. These healthcare professionals participated in “medicalized killing,” i.e., medically supervised killing in the name of healing [1]. They played a key role in genocidal projects that perceived mass murder as a therapeutic imperative [1]. These healthcare professionals were not forced to partake in these atrocities. More than half of the German physicians and nurses voluntarily joined the Nazi Party relatively early [2,3,4,5]. Those who opted out of the genocidal programs were not punished [6,7]. Moreover, medical scientists not only executed but also initiated and designed pivotal elements of Nazi policy [8]. It should be noted that Nazi healthcare personnel did not act in an ethically-free vacuum: a code of medical ethics existed prior to World War II [9].

Conversely, much can be learned from Jewish and non-Jewish doctors and nurses, who worked under dire conditions, including hunger, disease, and lack of supplies, to treat Jews. Jewish medical professionals worked within ghetto healthcare systems to deliver care, maintain sanitation, and contain epidemics, such as typhus and typhoid [10,11]. Moreover, there is great value in learning about Holocaust survivors, the trauma they experienced and its implications, as well as their resilience during and after the Holocaust. Addressing these topics provides an opportunity to enhance students’ understanding about providing optimal care that is both sensitive and tailored to the special needs of Holocaust survivors and other patients who have undergone severe trauma.

Awareness has increased over the recent years regarding the need to teach students in healthcare professions about this critical period in the history of medicine and nursing [9,12,13,14]. Nevertheless, the majority of nursing and medical schools do not include Holocaust and genocide studies in their curriculum [5,15]. Furthermore, existing curricula about the Holocaust most often focus on physicians and nurses who collaborated with the Nazi regime and rarely examine the functioning of the Jewish and non-Jewish physicians and nurses who treated Jews during the Holocaust or Holocaust survivors.

The aim of the present study is to help close these pedagogical and research gaps by analyzing a requisite curriculum on the Holocaust and genocide that is taught in a graduate nursing program. The focus of the current article is on the curricular components related to the work of Jewish healthcare professionals during the Holocaust, as well as Holocaust survivors, their treatment today, and the relevant implications for the current treatment of other patients.

## 2. Literature Review

### 2.1. Jewish Medical Staff during the Holocaust

Jews in ghettos and labor camps developed healthcare systems under inhumane conditions. In the Warsaw ghetto, for example, the healthcare system included several clinics, a laboratory, two hospitals, and an underground medical school [16,17]. The Kovno and Lodz ghettos had laboratory facilities; the Vilna ghetto had a preventative health program. These healthcare systems served the entire ghetto population, not just those able to pay [10,16]. Doctors in the ghettos also conducted research on typhus and hunger, as these conditions are much less common in “normal” circumstances. Many medical initiatives were documented and found after the war, as Jewish doctors recorded them to bear witness to what happened in the ghettos. These studies were later published and are still used today [11,17].

Establishing a healthcare system was an act of resistance [18]. The Nazi attack on public health in ghettos, such as deliberate overcrowding, lack of potable water, and lack of medical supplies, was part of the plan to exterminate Jews. The spread of disease, such as typhus and typhoid, also perpetuated the myth that Jews were disease-ridden and served as an excuse to crowd them into the ghetto, becoming a self-fulfilling prophecy [17]. Thus, healthcare workers fought battles on two fronts—against the Nazis, and against disease [10,11,16].

### 2.2. Ethical Dilemmas Faced by Jewish Healthcare Personnel during the Holocaust

The practice of Jewish medical professionals during the Holocaust demonstrates their commitment to the underlying principles of medical ethics and how they kept their identity as healers while facing enormous ethical dilemmas [10,19,20]. Wasserman and Yoskowitz described the actions of several Jewish physicians during the Holocaust, highlighting how a physician should act and their responsibility towards the patient [20]. Dr. Mark Dworzecki, for example, documented the atrocities he witnessed in the Vilna ghetto and the labor camps to which he was sent, feeling that it was his duty to bear witness. Dr. Viktor Frankl, when serving as the head of the neurology department of the Rothschild Hospital in Vienna, actively participated in attempts to rescue Jewish youths with mental disabilities from being sent to Hartheim Euthanasia Center. Later, as the head psychiatrist at Theresienstadt, Dr. Frankl helped inmates find purpose and a reason to live. Offer detailed the devastation felt by Dr. Adolf Polisiuk, who was forced to take part in the selection during deportation to Treblinka, and how he worked to console both his patients and colleagues being deported [11].

Perhaps one of the best-known examples of Jewish medical professionals facing ethical dilemmas was Dr. Gisella Perl, a gynecologist who worked in the infirmary in Auschwitz. Since abortions were forbidden and pregnancy meant either death or medical experimentation of the mother at the hands of Nazi doctors, Dr. Perl would hide pregnant Jewish women as pneumonia patients. She would perform secret abortions at night or deliver and kill the infant after birth. While Dr. Perl has stated that performing those abortions and deliveries haunted her, she also knew that allowing a woman to go full-term or allowing those infants to live meant certain death for both mother and child [20,21]. In another instance, Dr. Perl was ordered to take blood from all patients with a fever in order to diagnose typhoid. This would have meant certain death for any patient found positive. Instead, Dr. Perl and her staff took blood samples from one another, saving the lives of the ill [20].

Medical professionals needed to make tough decisions with regard to their patients, and these decisions could mean the difference between life and death. One Jewish pharmacist, assigned to work at the infirmary in Auschwitz, was given twelve antibiotic tablets with which to treat patients. This forced him to make decisions on which patient would benefit most from the treatment. In a sheer act of bravery, this pharmacist requested additional tablets, which were supplied, thus enabling him to treat more patients [22].

Nurses working in concentration camps described feeling helpless to truly help prisoners. Even when the nurses could help their patients to recover, these patients might later be murdered in extermination camps. Those in extermination camps had even fewer supplies, if any at all, and most care consisted of attempting to lower fever and treating patients with compassion. However, the work provided these professionals with a purpose, and enabled them to preserve their own humanity [22].

### 2.3. Treating Survivors

Holocaust survivors experienced extreme trauma during World War II, which affected their physical and mental well-being long after the war ended. Studies have found that survivors have poorer physical and mental health outcomes at old age compared to non-survivors. These include a higher incidence of certain diseases including cancer, and chronic diseases such as diabetes, hypertension, and metabolic syndrome [23,24,25]. Some studies found no difference in physical health, but differences in well-being and functionality [26]. Authors showed that survivors are more likely to suffer from depression and post-traumatic stress disorder (PTSD), while others found a higher prevalence of anxiety and sleep disorders [27,28,29].

The majority of survivors alive today were children during the war. These survivors experienced this trauma, which included persecution, starvation, and separation from parents and family, during their formative years, and their experiences affected health outcomes later in life. For example, child survivors of the Holocaust reporting poor post-war quality of care were more likely to report poorer well-being in their older age. Young children and children who did not lose their parents during the war were less likely to experience PTSD. Rates of PTSD varied between 37% and 55% among survivors who were under 18 years of age [30,31,32].

### 2.4. Resilience

While there is no consensus as to a definition, scholars agree that ‘resilience’ includes traits that enable an individual or group to overcome adversity [33,34]. Holocaust survivors have been found to be more resilient than non-survivors. It is likely that survivors who experienced extreme conditions during the war developed coping abilities which rendered them more resilient. However, it is also possible that the survivors’ resilience enabled their coping strategies. Indeed, children who survived the war demonstrated resilience by becoming stable and well-adjusted adults, despite their traumatic early childhood [35]. Survivors who managed to adapt, demonstrate industriousness, and maintain optimism have a high level of resilience [36].

A meta-analysis of over 12,000 participants found that survivors had a higher likelihood of post-traumatic stress symptoms and psychopathological symptoms compared to non-survivors, but there were no significant differences in other measures, such as cognitive function and adaption. The authors posit that the lack of difference between the groups was due to the combination of resilience and utilization of defense mechanisms among survivors, which enabled them to separately compartmentalize their experiences during the Holocaust from day-to-day functioning [37]. Shmotkin et al. also found that the majority of Holocaust survivors are able to lead successful lives [38]. However, aging, which can bring with it poorer health, and the need for hospitalization or nursing homes, can have a negative impact on resilience.

### 2.5. Teaching Biomedical Ethics Lessons from the Holocaust

Many aspects of modern medical ethics are based on the Nuremberg Code, which was created as a response to the horrific actions of Nazi medical personnel during the Holocaust. Despite this, however, very few medical and nursing schools teach their students about the roles played by healthcare personnel during the Holocaust. A survey conducted among Canadian and American medical schools found that only 16% reference the role of physicians during the Holocaust during ethics classes [15]. In Australia, a survey of nursing, midwifery, and medical schools found that few taught about the Holocaust and only several more referenced the Holocaust during research ethics courses [5].

In recent years, more attention is being paid to the importance of including lessons related to ethics during the Holocaust when teaching medical ethics [9,12,13,14,15,39,40]. The Autonoma University in Spain, for example, has sent medical students to Poland within the framework of an elective, interactive course for their students [41]. However, these educational initiatives are far from commonplace and their scope is limited. According to Ben-Sefer and Sharon, many students find it difficult to understand the relevance and implications of this topic to their lives and today’s world; hence, the topic needs to be viewed as something to be remembered (my own story) rather than as part of history (someone else’s story) [42]. Furthermore, the primary focus of the existing curricula is on the role played by Nazi physicians and nurses [12,39,43]. The role played by the Jewish health professionals is rarely studied. Issues related to the Holocaust victims and survivors are also usually ignored despite their relevance in understanding contemporary questions and topics around medical and nursing ethics and practice. This article aims to bridge this gap.

## 3. The Academic Framework and Curriculum

This article analyzes the Holocaust and genocide curriculum at the Jerusalem College of Technology (JCT). Since 2019, this curriculum has been mandatory for all first-year students at the integrated MSN + NP program: Master of Science in Nursing + Nurse Practitioner in geriatrics or palliative care. All the students in this program are experienced nurses.

The curriculum is composed of three elements: an academic course, a study day at Yad Vashem, the World Holocaust Remembrance Center in Jerusalem, and a study day in the Ghetto Fighters’ House Museum. The course includes lectures, group discussions, film analysis, survivor testimony, interactive peer learning, and students’ presentations of their concept analyses. The course lecturers include nurses, physicians, historians, social scientists, and a lawyer, each from various universities. The authors initiated, led, and coordinated the program. To date, 87 students have participated in the full program. In addition, dozens of other undergraduate and graduate students, as well as faculty and staff, have attended lectures that were open to the entire JCT community.

The curriculum examined four main aspects of the Holocaust and genocide. The first pertains to the Jewish physicians and nurses during the Holocaust, the dedicated treatment they provided under impossible conditions, the tremendous moral dilemmas and conflicts they faced, as well as their research and teaching. The nursing students learned about leadership in times of crisis, focusing primarily on women leaders. The courage and professionalism of the Jewish healthcare providers inspired the students and led to discussions on contemporary issues that will be elaborated in the Results section of this paper.

The second aspect relates to Holocaust survivors and lessons that can also be learned about other groups of patients. The students learned about the physical and mental morbidity, as well as the resilience, experienced during the Holocaust and those which currently characterize Holocaust survivors. They received professional tools to better understand and treat Holocaust survivors and other traumatized patients. The students also examined the survivors’ testimonies at Eichmann Trial (1961) and how the survivors were socially constructed by Israeli society over the years. Drawing on the historical events, the students critically analyzed and presented related concepts in class, such as hope, coping, dehumanization, dignity, human rights, resilience, resistance, and trauma, and how these concepts can manifest in their nursing praxis.

The third aspect relates to the collaboration of German healthcare professionals with the Nazi regime. This included a visit to the exhibition “Deadly Medicine: Creating the Master Race” at the Ghetto Fighters’ House Museum. This exhibition, which was curated by the United States Holocaust Memorial Museum, presents the Nazi “science of race” and various activities from 1933 to 1945 meant to “cleanse” the German society of people viewed as threats to the nation’s “health”. It also traces this ideology’s roots to the early 20th-century eugenics movement in other countries. The student nurses discussed the symbiosis between healthcare professionals and the Nazi state, and these professionals’ engagement in designing, legitimizing, and administering Nazi “racial health” policies—from the mass sterilization of “hereditarily diseased” people until the murder of millions of people [1,8,39]. We analyzed the acts of nurses who chose to participate in the T4 “Euthanasia” program as a case study. The students also examined the “medical ethics” under the Nazi regime in a guest lecture of Dr. Tessa Chelouche.

The fourth aspect of the curriculum focuses on questions of social denial and the mechanisms that allow the indifference of bystanders, such as those who resided in the areas surrounding the ghettos and concentration camps, as well as the denial of past atrocities, such as the Armenian genocide. The students also learned about other genocides such as those which occurred in Rwanda, Bosnia, and Darfur and the international reaction (or lack thereof) to those events.

The course emphasized the relevance of these issues to contemporary professional, bioethical, moral, and societal issues. For example, students inquired whether concepts such as eugenics exist in today’s healthcare system. They were also disturbed by the realization that indifference characterizes our society and that they themselves are indifferent to contemporary cases of structural violence, wars, and humanitarian crises presented by guest lecturer Prof. Yair Auron. We discussed current issues of racialization, racism, xenophobia, discrimination, marginalization, and structural vulnerability.

This article focuses on the curriculum’s first and second aspects: the perspective of Jewish nurses and physicians, as well as the perspective of the Holocaust survivors, and how the learning of these issues affected the graduate students. Although it is beyond the scope of this article to elaborate on the third and fourth aspects of the curruiculum, we propose that an integrative and holistic program that covers all of these perspectives is efficient in realizing meaningful educational objectives.

## 4. Materials and Methods

In the program’s pedagogical process, the role of nurses as critically reflective practitioners was emphasized [44,45,46]. Reflective practice can make a significant contribution to nursing; however, it has multiple definitions and interpretations [47]. Galutira defined reflection in nursing as “a detailed exploration of a clinical situation or experience which includes an analysis of personal feelings, thoughts, and actions or behaviors. It entails cognitive activities such as description, critical analysis, evaluation, and planning” ([48] p. 52). Reflection is an active, dynamic, and transformative process that allows practitioners to make sense of an experience or a clinical situation, learn from it, critically examine “self,” change their perspectives and behaviors, and improve future practice [47,48]. It entails theorizing practice, ensuring that the nursing work is informed by a knowledge base open to scrutiny and challenge and a clear value base [46]. Reflection includes an interplay of the cognitive dimension, the affective dimension, and the action dimension that can potentially help nurses make a difference in the world [47].

Students were encouraged to be reflective in their nursing practice, their in-class and field learning, as well as the connection between them. Students were encouraged to be involved in both reflection-in-action and reflection-on-action [45], by writing reflective accounts during the course and sharing their reflections in class. Reflection-in-action means “thinking and drawing on our professional knowledge base while engaged in practice” ([46] p. 12). This process “serves to reshape what we are doing while we are doing it” ([49] p. 26). Reflection-on-action refers to retrospective critical thinking: “stepping back and drawing out the learning after having engaged in practice” ([46] p. 12). This process constructs and reconstructs events in order to develop oneself as a critically responsive and sensitive practitioner and person [47]. It also informs the subsequent reflection-for-action that involves planning and thinking through before engaging in practice [46].

The students’ reflective writing helped them reconceptualize and see anew their daily practices that they often have no time to critically explore. Importantly, reflection was found to be an effective method that enables nurses “to find their own voice,” develop open-mindedness, critical awareness, and doubt, as well as to see what others tend to ignore or deny, and ultimately challenge oppression and promote social justice and human rights in their practice [47,50,51]. These abilities are highly relevant to the present curriculum of Holocaust education and therefore this method is suitable for this pedagogical program.

To understand the students’ perspective and learning process, this article presents parts of the reflective accounts written by the students. These reflections were analyzed inductively by using qualitative content analysis and Grounded Theory. This included an iterative search procedure for expressions and ideas [52]. The interpretative narrative analysis included several stages. Following a holistic reading of the raw data, we conducted open coding that included an initial division of the data into many thematic categories. We subsequently identified connections between these initial categories, refined them, and combined similar categories. We chose core categories that comprised the meaningful thematic clusters around which we organized the findings [52]. Finally, after we formed the conceptual descriptions and explanations, we reread the raw data to confirm that the analysis did not depart from the data. Students’ texts were anonymized and pseudonyms were used for the students’ names. The cited students consented to the publication of quotations from their texts.

## 5. Results: The Effect of the Studies on the Nursing Students

### 5.1. Jewish Physicians and Nurses during the Holocaust

The students learned about the Jewish physicians and nurses who not only continued to provide healthcare but also adhered to their research and teaching tasks in the subhuman conditions of the ghettos and concentration camps. Thus, for example, Dr. Miriam Offer and Dr. Shai Feuering described the activities of around 800 healthcare professionals who continued working in the Warsaw ghetto. The lectures provided an in-depth exploration into a few key figures and the moral and professional dilemmas which they experienced, among them Adina Blady Szwajger, a physician who worked at the Children’s Hospital in the Warsaw ghetto [53]. In the summer of 1942, a few days before the “Grossaktion Warsaw,” which included the deportation and mass murder of Jews, she and another nurse administered lethal doses of morphine to children to prevent them from suffering and agonizing death in the overcrowded Holocaust trains and the extermination camps.

This case enables a discussion of mercy killing and other impossible ethical and existential quandaries and predicaments under extreme adversity [54]. Halpin, for example, asserts that although Blady Szwajger defied the Hippocratic oath to do no harm, the philosophical theory of utilitarianism—i.e., considering what action will achieve the greatest good for the greatest number of people—can explain her act: “Blady Szwajger was striving to create the least amount of harm by preventing a greater amount of suffering she felt certain the children would otherwise have to endure. Arguably, it is a complex case of facing not only an ethical dilemma but also the forces of the human condition of empathy, fear, anxiety, foreboding and other emotions” ([55] p. 350). Thus, this and other cases can be used for teaching not only contested and crucial issues such as euthanasia, which are still debated today, but also basic concepts in moral philosophy, such as deontology versus utilitarianism and Arendt’s concept of “the human condition” [56]. Moreover, the situations faced by the Jewish healthcare professionals can teach us about “choiceless choices,” or “crucial decisions [that] did not reflect options between life and death, but between one form of abnormal response and another, both imposed by a situation that was in no way of the victim’s own choosing” ([57] p. 72).

Students specializing in palliative care analyzed the case of Adina Blady Szwajger and debated the significance of this type of mercy killing. One of the students, Rose, expressed her admiration for “this physician’s and nurse’s valor […], although this type of act is considered a transgression against the current principles of medical ethics”. Another student, Mika, expressed the opposite view, stating that she was “profoundly shaken by the story. […] I would have chosen either life or death, but I would have attached my own fate to that of the children”. Mika explained her opinion that if Blady Szwajger decided to kill her patients, the moral choice would be to commit suicide and die along with these children. If Blady Szwajger decided to try and survive, she also had to give her children a slim chance of survival. Other students reflected on the case of Blady Szwajger as a way of conducting an in-depth examination of their own professional practices and ethical dilemmas. Talia openly shared the following:

“I think about her and somehow I think about myself and the impact that five years of nursing in a pediatric intensive care unit left on me. […] There are some dilemmas that are never resolved when you treat children in the most critical conditions. More than a few times we had to treat children whose condition was fatal […], even while we knew that it would be of no avail. But I kept thinking to myself that I don’t really know […] whether the treatment I am providing holds a sliver of hope or only causes further suffering to the patient. There were several times when I was caring for a child in a fatal condition and at the same time was praying that death would come and thus end the child’s suffering. On those occasions, I would ask myself: ‘What kind of a nurse am I who prays for her patient to die?’”

The course also examined the role of non-Jewish medical professionals, such as prisoner doctors, who offered assistance in the camps. One of the guest lecturers in the course was the Former Chief Rabbi of Israel, Rabbi Yisrael Meir Lau, who is also a Holocaust survivor. In his lecture, he told the story of a non-Jewish, communist, Czech prisoner physician who saved his life in Buchenwald concentration camp in January 1945. Indeed, it is the positions of power that are significant in learning about the contraventions of medical ethics, rather than the fixed historical identities of German doctors versus Jewish victims.

### 5.2. Treating Holocaust Survivors

During the course, the students learned about the types of physical and mental morbidity experienced during the Holocaust, and those which currently characterize Holocaust survivors. In Israel, there are currently 187,500 Holocaust survivors, about 25% of them are living below the poverty line [58]. Dr. Laurie Glick presented data about late organic morbidity among Holocaust survivors and their difficulties in physical and cognitive functioning. She provided details about ways to provide nursing care and support for this population and also how to best communicate with them. Given that today’s survivors were children during the Holocaust, Dr. Haya Raz focused on the experience of growing up in a time of crisis and its impact in the long-term, referring to theories such as terror management theory of social behaviour [59].

The students noted that studying these topics helped them to gain a better understanding of their patients who are Holocaust survivors, and how to appropriately treat them. They mentioned that many of their geriatric and palliative patients relive the trauma of the Holocaust and that the course provided them with ways to cope with this phenomenon. Mika explained: “I work at an oncological hospice and oftentimes I see Holocaust survivors who reexperience the reality of the Holocaust during their moments of confusion”. Learning about this population through the course helped Mika “understand what they were going through, their fears, the family dynamics, and when I need to call on an authority figure (i.e., a social worker or psychologist) to intervene”.

Following this course, the nurses adopted a new perspective which was more respectful and empathic towards Holocaust survivor and post-trauma patients. Tamar described the following: “Many times we find that Holocaust survivor patients are more verbally—and sometimes physically—aggressive. Our visit to Yad Vashem made me realize that we mistakenly interpreted this behavior as an expression of their lack of respect toward us. Sometimes the staff is too self-absorbed instead of focusing on the patient, who had experienced trauma and humiliation and had lost loved ones. Then instead of calming the patient with empathy and care, we are concerned with whether or not the patient treats us with respect”.

Tamar also offered some practical suggestions: “We should create support groups for patients who are Holocaust survivors. […] During the anamnesis of a newly admitted patient, we should note whether the patient is a Holocaust survivor and set up a meeting with the social worker on a routine basis. […] Nursing schools should dedicate at least a week to teach about the Holocaust and its relevance to healthcare treatment”.

### 5.3. Resilience and Hope

The course also emphasizes the resilience and hopefulness expressed by the Jews during the Holocaust and by the survivors. Alma, one of the students, wrote about the survivors’ resilience: “I am in awe of the strength it took to survive on a daily basis, even after the Holocaust. How did people who had suffered so much find the strength to build a new life? […] How did they cope? How did they get up in the morning and continue day after day? And even beyond the ‘how,’ why did they do it? What for? […] I am interested in the practical and mental ways that people manage to recruit the strength needed to find and maintain resilience”.

Students wrote about the meaning of hope in the context of the Holocaust, as well as hope among patients and their current staff members. Following a discussion on the meaning of hope during the Holocaust, Talia commented: “I understand that what motivates them all is the sense of hope, the hope for better days. […] Again, I think about my work in the pediatric intensive care unit, about the little girl with an enormous tumor in her liver, after 14 h of surgery. […] We cared for her as best we could, even though we weren’t really sure if she would be able to recover. But we were wrong to doubt because recover she did—incredibly well. Her mother never stopped believing even for a moment and was constantly hopeful that she would overcome. Yet I think about many other parents who were also hopeful, but their children did not survive. Even when you are in your own personal hell, in the darkest of times, hope can blossom. Sometimes it will save you and sometimes it won’t. Either way, it gives you the strength to cope. I find that heroic”.

### 5.4. Women Leaders

The course, in which all the students were women, also addresses the issue of leadership and gender. Guest lecturer Prof. Dina Porat talked about Jewish women who were leaders during the Holocaust and analyzed the factors that led women to take on a position of leadership. Dr. Sharon Geva examined the ways in which female Holocaust survivors were perceived in Israeli culture and society over the years. For example, Geva found that from 1948 (the establishment of the State of Israel) until 1961 (the Eichmann Trial), the spectrum of women perceived as Holocaust heroines was quite broad, with the main criterion being their willingness to self-sacrifice, like the fighters and mothers in the Holocaust. Geva disproved the common belief that until 1961 the Holocaust was not part of the public discourse in Israel. She demonstrated that female Holocaust survivors were not silenced or ignored but were prominent, active, and discussed their experiences in public [60]. The students found these historical figures to be inspirational and viewed this portion of the course as part of their socialization and preparation for a position of leadership as nurse practitioners. This is significant given the gender balance in nursing.

Orit, a nursing student, commented: “Women have replaced men as committee representatives, as laborers, rescuing children, joining resistance movements […]. In the nursing profession, women have a great deal of power. […] Yet, sometimes I think we do not sufficiently appreciate our abilities”. The course empowered the nurses to appreciate their abilities and become conscious leaders in the healthcare system. It was also a central and effective element in a broader educational process that aimed to increase the nurses’ civic and community engagement [44,61,62,63].

### 5.5. The Hospital as a Total Institution

Students noted that the unit on Holocaust studies led them to a better understanding of the difficulties experienced by patients when they are admitted to the hospital and stripped of their former identity and personality. Indeed, the students spoke about the characteristics of the hospital as a total institution [64], a concept that was already familiar to many. Yet, their understanding became more experiential and emotional after studying the most extreme example of dehumanization. The students said that the course helped them internalize the importance of treating each patient on a personal level. Sarah’s words demonstrate this realization: “In the field of nursing, we work in a heavily burdened system, which often has no grasp of the individual. […] A patient is admitted, stripped of his or her clothes, and dressed in a uniform hospital pajama. […] And you must abide by the departmental schedule”.

Leah added: “Do I refer to patients by their names or professions? No. When talking to my colleagues, I refer to ‘the patient in bed number two in room three.’ The realization that patients should be treated as human beings and not as numbers is not new to me […]; however, our visit to Yad Vashem heightened this understanding so that it reached an emotional level”.

The students especially attributed this understanding to cases of patients who had “lost their identity,” such as patients on respirators or patients with dementia. They claim that they had a more acute awareness of the need to treat these patients as a whole person, remember their identity and their past, and treat them respectfully. This lesson was especially important given that the students were specializing in geriatric nursing or palliative care and consequently encounter patients in these conditions on a daily basis.

Following an intensive day of study, Kathy wrote the following: “‘Every person has a name’—I’ve decided to make this my motto from now on. […] Many of my patients, regrettably, have lost their sense of identity. […] Patients who are chronically on respirators, disabled, with dementia, multiple sclerosis, or muscular dystrophy; people who after a stroke have no way of communicating with the world. Unfortunately, more than a few staff members treat them like the shell of the person they once were. […] When approaching these patients, we should talk to them, explain what we are going to do, ask how they are doing, even if they are incapable of answering; smile at them. […] We want to avoid treating people as numbers, dehumanizing them as the Nazis did, stripping them of their identity and their respect”.

Learning about the Holocaust survivors’ mental condition at the end of the war, when they were released from the camps, helped the students understand how to treat patients who have undergone physical or mental trauma. Mika made the following comment: “When I saw the photographs of the survivors wailing and crying and not at all happy upon their release, I thought of the geriatric patients, or of the patients who have completed a long and difficult period of rehabilitation, after which they are simply told to go home. They are completely in shock; they don’t know how to live their daily life, they have no strength, they don’t understand what they are supposed to do, and sometimes there is no one around to care for them. We need to understand this sense of helplessness and try to assist them, find them a supportive framework, until they are able to function independently again. Returning to daily life after a physical or mental trauma is incredibly difficult and frightening”.

### 5.6. The Pedagogy of Learning History from a Personalized Perspective

The pedagogical approach we used in this course was to emphasize the personal and emotional connection that the students felt toward the contents of the course. We encouraged the nurses to constantly exercise reflective observation. Some of them noted that this course was first time they experienced reflective writing. We also encouraged the students to think about their patients, just as the study of this period in history was focused on cases of individuals. Notwithstanding its effectiveness, this approach has its difficulties as it tends to evoke emotions in students such as pain and sorrow, guilt and regret.

Yael’s impressions written after the visit to Yad Vashem demonstrate that students perceived their personal lives, their professional lives, and the study of the Holocaust to be intimately connected:

“On the personal level, this took me back to a time in my life, about six years ago, when I stepped away from a violent relationship. […] One day I found myself with my five children in a shelter for battered women. The entire year we lived without any belongings, without a home; the children were my entire world and I watched over and protected them like a lioness with her cubs. A year later we started over in a different city, far from anything familiar. I had nothing; I had to start rebuilding my entire life, while experiencing moments of breakdown and loss. So, when I stood in front of the exhibit of shoes at Yad Vashem, I was overwhelmed with emotion. […] On the way home, I thought about those mothers, the price they had to pay, trying to protect their children, and about children who had lost their entire world in a flash. I thought of the horrors and again I saw the faces of my brave patients. I had found it difficult to understand their reactions to light, darkness, touch, the sound of the siren on Memorial Day; every small shift was difficult for them. And suddenly, I understood. They too were emotionally overwhelmed, by the slightest changes, which to us seemed trivial and meaningless. I suddenly remembered Eva, who begged us not to throw out the old breadcrusts, and we did as she asked. […] The experience of the visit to Yad Vashem was very strong and emotional for me personally; it had implications for my daily activities. I understood the need to illuminate the personal aspects of each patient in a more sensitive manner, and to accept their unusual behaviors with greater compassion”.

Yael was able to find a new sense of compassion towards her patients who are Holocaust survivors or those who have experienced trauma, based on a connection to a personal crisis that she experienced, and she drew conclusions from this experience and applied them to her nursing care practices. The three dimensions, that is, the history of the Holocaust, her private life, and her nursing practice, were simultaneously evoked during her visit to Yad Vashem. It is important that the teaching staff in this course adopt an increased awareness and closely follow the students’ reactions as they experience these complex emotions. It is essential to conduct a class discussion, during which each student, in turn, is given the opportunity to share personal emotions and experiences, which are then validated by the others. This allows the students to get the support, empathy, legitimation, and advice from the peer group and teaching staff. The exercise of reflective writing also helps the students process the experience, and it is recommended to encourage them to have someone with whom to reflect.

These recommendations also respond to another major drawback of the reflection method. Research found that students who are engaged in reflection are often overly critical of their performance. This might yield negative feelings that can form barriers towards learning, distort perceptions, and undermine the will to persist [65]. This phenomenon was evident, for instance, in the aforementioned quote from Talia who asked, “What kind of a nurse am I who prays for her patient to die?” This kind of self-criticism, which is often embedded in the reflective approach, can be emotionally challenging, and the group support is useful in coping with these feelings.

Ada was reminded of her own childhood of poverty when she saw a photograph of a hungry child at the exhibit at Yad Vashem. She told us about her childhood with a father who was subjected to drug and alcohol abuse and who was violent towards her mother and brother, both of whom had intellectual and developmental disabilities. Ada looked at the photograph of the hungry child and was reminded of her own childhood: “Closed inside myself, my stomach gurgling, feeling hungry, again and again […]. For years, only hope and my imagination saved my soul from devastation”. She saw the photo and “It took me back to such a long time ago, that photo of the skinny child in the ghetto dressed in tatters, lying in the fetal position, enormous eyes. I could only hope he was using his imagination! Imagining a better world. […] Did imagination give him the hope he needed, something to hang onto, so as not to fall into the abyss?”

One can ask whether imagination could really have saved the child in the photograph from the abyss, or if Ada projected her own experience on to this child. Ada’s reflection epitomizes both a vital strength and a pitfall of the reflection method. The self-observation and affective dimension of this method allow a deeper identification with and internalization of the course materials. The reflective approach helped Ada rethink the concept of hope that was discussed in class from a personalized perspective. At the same time, however, scholars have shown that a common drawback of using personal forms of critical reflection is that students can slide into isolated thinking and self-absorption and become inward looking, allowing self-inspection to dominate their work and their thinking [45,65,66].

A recurring theme in students’ reflections was the need to share with others (at work and at home) the experiences and the insights they had gained and to continue discussing what they had learned. The recurrence of this theme is an indication of the necessity to encourage group discussions beyond the classroom and to create a suitable platform for this purpose. Rebecca told the following regarding the visits to Yad Vashem and the Ghetto Fighters’ House: “Our trip to the two sites left their mark on me; I couldn’t stop thinking and I shared this with my coworkers. In the days that followed our visit, we had several discussions in the department on various topics related to the Holocaust. […] The subject led to spirited debates and discussions among my colleagues”.

Orit also mentioned having follow-up discussions in broader circles: “Every time I come home [after our class] I am full of energy, excited, and eager to tell the whole world what I learned that day. […] Learning about the Holocaust, about the practice of medicine during the Holocaust, the activities of German physicians and Jewish physicians changed my view of providing healthcare and nursing services. I see my patients in a new light. Today I am a better nurse”.

## 6. Conclusions

Teaching the Holocaust in nursing schools is uncommon. When these studies exist, they often focus on the collaboration of physicians and nurses with the Nazi regime. The current study presents a comprehensive requisite curriculum, which includes a review of the impressive healthcare system established by Jewish physicians and nurses in the ghettos and the terrible and often irresolvable ethical quandaries, conflicts, predicaments, and “choiceless choices” [57] they faced as they attempted to deliver healthcare in subhuman conditions. The course described throughout this paper also provided graduate-level nursing students with professional, up-to-date, and evidence-based tools for optimal treatment of Holocaust survivors, post-trauma patients, and others. The students reported that the course had provided them with tools to act professionally and empathetically while demonstrating greater patience and sensitivity to the patients’ identity and past experiences. The notion that learning about a patient’s background is crucial to their care can also be applied to other situations of atrocities, and even to everyday life. Furthermore, the students arrived at a more profound understanding of the meaning of a total institution for hospital patients. Importantly, studying the Holocaust encouraged the student nurses to become “witnessing professionals” who reveal, contest, and combat the “malignant normality,” i.e., the underlying dangerous assumptions and narratives that prevail in their society [67].

The pedagogical approach adopted for this course emphasized the personal stories, difficulties, and dilemmas of the nurses and physicians in the Jewish ghetto, as well as those of current patients who are Holocaust survivors. This personalized approach contributed to experiential learning and the ability to internalize the material studied on an emotional level. It should be noted, however, that this intensive involvement in studying difficult issues requires close and constant awareness and accompaniment on the part of the teaching staff. Notwithstanding, while engaged in this course, many of the students felt hopeful and developed a sense of agency as conscious leaders capable of creating a positive and significant change in their environment.

In light of these findings, we suggest that the study of the Holocaust and genocide be incorporated as a requisite component in every nursing program worldwide. The lessons learned from the Holocaust are universal, and the proposed curriculum is highly relevant to any country and any national and social context. The study of these issues should not be restricted to Israel or countries where Holocaust survivors reside, as the ethical and professional issues discussed in this course are significant for any healthcare worker and for treating vulnerable patients.

The two major centers for Holocaust teaching in Israel, namely, Yad Vashem and the Ghetto Fighters’ House, offer an excellent pedagogical infrastructure for experiential and multisensory learning. These two institutions, not dissimilar to other museums that deal with this topic, offer opportunities for distance learning and guidance for international audience, services that have been upgraded in the era of the COVID-19 pandemic. Finally, there is no reason that the studies should be solely offered to students; rather, they should be accessible also to healthcare professionals who have already completed their studies, possibly as part of their professional development. This course presents an opportunity to also affect those who are already in the field.

## Data Availability

Not applicable.
